# Alveolar compartmentalization of inflammatory and immune cell biomarkers in pneumonia-related ARDS

**DOI:** 10.1186/s13054-020-03427-y

**Published:** 2021-01-09

**Authors:** Inès Bendib, Asma Beldi-Ferchiou, Frédéric Schlemmer, Mathieu Surenaud, Bernard Maitre, Anne Plonquet, Guillaume Carteaux, Keyvan Razazi, Veronique Godot, Sophie Hüe, Armand Mekontso Dessap, Nicolas de Prost

**Affiliations:** 1 Service de Médecine Intensive Réanimation, Hôpitaux Universitaires Henri Mondor, Assistance Publique-Hôpitaux de Paris, 51, Avenue du Maréchal de Lattre de Tassigny, 94010 Créteil Cedex, France; 2 Groupe de Recherche Clinique CARMAS, Faculté de Santé de Créteil, Université Paris Est Créteil, 94010 Créteil Cedex, France; 3 Université Paris Est Créteil, INSERM, IMRB, 94010 Créteil, France; 4 Département d’Hématologie et d’Immunologie biologiques, AP-HP, Groupe Hospitalo-Universitaire Chenevier Mondor, 94010 Créteil, France; 5 Unité de Pneumologie, Service de Médecine Intensive Réanimation, Hôpitaux Universitaires Henri Mondor, Assistance Publique-Hôpitaux de Paris, Cedex 94010 Créteil, France; 6 INSERM U955, Equipe 16, 94 000 Créteil, France; 7 Vaccine Research Institute, 94 000 Créteil, France; 8 Faculté de Médecine, Université Paris Est, 94 000 Créteil, France; 9 INSERM U955, 94 000 Créteil, France

**Keywords:** Respiratory distress syndrome, Adult, Pneumonia, Cytokines, HLA-DR antigens, PD-1

## Abstract

**Background:**

Biomarkers of disease severity might help individualizing the management of patients with the acute respiratory distress syndrome (ARDS). Whether the alveolar compartmentalization of biomarkers has a clinical significance in patients with pneumonia-related ARDS is unknown. This study aimed at assessing the interrelation of ARDS/sepsis biomarkers in the alveolar and blood compartments and explored their association with clinical outcomes.

**Methods:**

Immunocompetent patients with pneumonia-related ARDS admitted between 2014 and 2018 were included in a prospective monocentric study. Bronchoalveolar lavage (BAL) fluid and blood samples were obtained within 48 h of admission. Twenty-two biomarkers were quantified in BAL fluid and serum. HLA-DR^+^ monocytes and CD8^+^ PD-1^+^ lymphocytes were quantified using flow cytometry. The primary clinical endpoint of the study was hospital mortality. Patients undergoing a bronchoscopy as part of routine care were included as controls.

**Results:**

Seventy ARDS patients were included. Hospital mortality was 21.4%. The BAL fluid-to-serum ratio of IL-8 was 20 times higher in ARDS patients than in controls (*p* < 0.0001). ARDS patients with shock had lower BAL fluid-to-serum ratio of IL-1Ra (*p* = 0.026), IL-6 (*p* = 0.002), IP-10/CXCL10 (*p* = 0.024) and IL-10 (*p* = 0.023) than others. The BAL fluid-to-serum ratio of IL-1Ra was more elevated in hospital survivors than decedents (*p* = 0.006), even after adjusting for SOFA and driving pressure (*p* = 0.036). There was no significant association between alveolar or alveolar/blood monocytic HLA-DR or CD8^+^ lymphocytes PD-1 expression and hospital mortality.

**Conclusions:**

IL-8 was the most compartmentalized cytokine and lower BAL fluid-to-serum concentration ratios of IL-1Ra were associated with hospital mortality in patients with pneumonia-associated ARDS.

## Background

The acute respiratory distress syndrome (ARDS) is the most severe form of acute hypoxemic respiratory failure and affects 10% of all intensive care unit (ICU) patients. Despite advances in patient management during the previous decades, hospital mortality of ARDS remains as high as 40% [1]. As most pharmacological interventions tested in ARDS yielded disappointing results [2–4], the identification of biomarkers of disease severity that would be potential therapeutic targets or allow for individualizing patient management has become a major area of research. Indeed, combining plasma biomarkers and clinical variables has been shown to improve mortality prediction in ARDS patients [5] and allowed for identifying subphenotypes with different clinical outcomes and therapeutic intervention responses [6, 7]. While blood has been the most common biological sample used to search candidate biomarkers, bronchoalveolar lavage (BAL) fluid is the closest sample to the site of injury and more accurately reflects the local lung environment [8], as illustrated by a pioneer study that identified BAL fluid—but not plasma—levels of IL-8 to predict ARDS development in at-risk patients [9]. In fact, no single biomarker obtained from blood samples has been shown to be consistently associated with outcomes in a recent systematic review [10]. This lack of association may be due to an alveolar compartmentalization of biomarkers during pneumonia-related ARDS.

Pulmonary infections account for the vast majority of ARDS risk factors [11] and are associated with septic shock in about 70% of cases. In patients with septic shock, a sustained decrease in HLA-DR expression on circulating monocytes [12, 13] was consistently associated with an increased risk of nosocomial infections [14] and a higher risk of death [14–16]. Programmed death receptor-1 (PD-1) is an inhibitory immune checkpoint receptor expressed on activated lymphocytes and myeloid cells, which participates to immune tolerance maintain [17]. Preclinical experiments using ARDS [18] models showed a survival benefit of PD-1 pathway inhibition, suggesting that PD-1 expression on immune cells could be an outcome biomarker in patients with sepsis [19–21] and ARDS [18]. Sepsis-induced defects in innate and adaptive immune cells were not only observed in blood but also in the lungs of patients dying from sepsis, illustrating that such immune alterations also occurred in situ, although the clinical significance of such regional alterations has not been established [22]. Monitoring blood monocyte HLA-DR expression has been previously used to guide targeted immunological interventions [23–25] and it has been speculated that the quantification of HLA-DR on alveolar monocytes [26] may enrich the identification of patients who might benefit from immunomodulatory interventions [16, 27, 28].

Better understanding the interplay of ARDS biomarkers between the alveolar and blood compartments seems a critical step to provide new insights into pathogenesis. In the current study, we aimed to assess in a prospective cohort of patients with moderate to severe pneumonia-associated ARDS: (1) the interrelation of ARDS/sepsis biomarkers in the alveolar and blood compartments, and (2) explore their association with clinical outcomes.

## Materiel and methods

### Study design

This prospective single-center observational cohort study was approved by the institutional ethics committee (Comité de Protection des Personnes Ile-de-France V, Paris, France, #13899). Consecutive patients diagnosed with pneumonia-related ARDS admitted to the medical ICU of Henri Mondor Hospital, Créteil, France, from January 2014 to December 2018 were eligible for inclusion in the study. Informed consent was obtained from all included patients or their relatives.

### Patients and data collection

All patients with moderate/severe pneumonia-related ARDS [11] were included consecutively with the following inclusion criteria: tracheal intubation and mechanical ventilation since less than 48 h; pulmonary infection diagnosed less than 7 days before ICU admission; bilateral pulmonary infiltrates on chest X-ray; a PaO_2_/FiO_2_ ratio ≤ 200 mmHg with a positive end-expiratory pressure (PEEP) ≥ 5 cm H_2_O. Non-inclusion criteria were as follows: age < 18 years; pregnancy; chronic respiratory failure requiring long-term oxygen therapy; Child–Pugh C liver cirrhosis; lung fibrosis; immunosuppression, SAPS II (Simplified Acute Physiology II score) > 90, irreversible neurological disorders, patients with withholding/withdrawing of life-sustaining therapies and profound hypoxemia (PaO_2_/FiO_2_ < 75 mmHg).

Control patients (i.e., non-mechanically ventilated patients free of ARDS or immunosuppression; *n* = 7) undergoing a bronchoscopy with bronchoalveolar lavage (BAL) and blood sampling as part of routine care were also included (Additional file 1: Table S1). None of the controls was receiving antibiotics at the time of BAL fluid and blood sampling.

Demographics, clinical and laboratory variables upon ICU admission, at samples collection time points and during ICU stay were prospectively collected. The initial severity of ARDS patients was assessed using the SAPS II [29] and the sequential organ failure assessment (SOFA) scores. Other variables included the use of adjuvant therapies for ARDS (i.e., neuromuscular blocking agents, nitric oxide inhalation, prone positioning, extracorporeal membrane oxygenation), the need for hemodialysis or vasopressors, the administration of corticosteroids, the number of ventilator- and organ failure-free days at day 28 and ICU mortality. The clinical endpoint of the study was hospital mortality.

ARDS patients received mechanical ventilation using a standardized protective ventilation strategy [30]. Other treatments, including neuromuscular blocking agents [31], nitric oxide inhalation [32], prone positioning [33] and extra-corporeal membrane oxygenation were administered depending on the severity of ARDS [34]. The prevention of ventilator-associated pneumonia followed a multifaceted program [35]; Sedation and mechanical ventilation weaning followed standardized protocols [36].

### BAL fluid and blood sampling

BAL fluid was collected and preserved undiluted from all ARDS patients during a bronchoscopy performed within 48 h of ARDS onset. BAL fluid samples were also collected from control patients. Concomitant blood samples were obtained in ARDS and control patients. During a standard flexible bronchoscopy, the bronchoscope was wedged within a bronchopulmonary segment. Four aliquots of normal saline (50 mL each) were instilled through the bronchoscope within the selected bronchopulmonary segment. After each aliquot was instilled, saline was retrieved using a negative suction pressure (BAL fluid return did not differ between ARDS patients and controls: median = 59 mL [first-third quartiles] [46–74] mL versus 80 mL [48–91], *p* = 0.40). BAL samples were filtered through a 100 μm cell strainer, centrifugated and BAL cells were then collected in phosphate buffered saline solution. BAL fluid cytology was performed by direct microscopy after centrifuging bronchoalveolar lavage fluid samples (12,000 revolutions for 10 min) and dying under the May–Grünwald–Giemsa staining. Total (quantified in cells/mL) and differential (i.e., percent of neutrophils, macrophages and lymphocytes) cell counts were measured as recommended [37].

Blood and BAL fluid samples were shipped at room temperature to the cytometry platform and analyzed within two hours. BAL fluid and blood samples were centrifuged and supernatants were stored at − 80 °C for subsequent analyses.

### Flow cytometry analysis

Blood and BAL fluid immuno-staining were performed as follows: 100 μL of whole blood or BAL fluid were incubated for 10 min at room temperature in the dark with the following conjugated-monoclonal antibodies: anti-CD3-AA750, anti-CD8-AA700, anti-CD279 (PD-1)-PC7 or isotype control, anti-HLA-DR-PB or isotype control, anti-CD14-ECD and CD45-Krome Orange (Beckman Coulter). For blood samples, red-blood cells were then lysed using VersaLyse Solution (Beckman Coulter). Washed blood and BAL fluid-stained samples were immediately acquired on a 10-multicolor Navios flow cytometer and analyzed with the Kaluza 2.1 software (both from Beckman Coulter). The gating strategy is depicted in Additional file 1: Figure S1 in BAL fluid (Panel A) and blood (Panel B). HLA-DR and PD-1 quantification were expressed in percentage of positive cells or mean fluorescence of intensity (MFI).

### Inflammation and endothelium/alveolar epithelium injury biomarkers quantification in BAL fluid supernatant

Cytokines were measured at distance using Luminex® multiplex bead-based technology (R&D Systems, Minneapolis, MN, USA) and a Bio-Plex 200® instrument (BioRad, Hercules, CA, USA), according to the manufacturers’ protocols. BAL fluid concentrations of 22 biomarkers, including inflammatory markers and cytokines/chemokines (interleukin (IL)-1Ra, IL-6, IL-7, IL-8, IL-10, IL-12/23p40, IL-13, IL-17A, interferon (IFN)-γ, tumor necrosis factor (TNF)-α, granulocyte–macrophage-colony stimulating factor (GM-CSF), RANTES, CXCL10, Serpin E1), endothelial injury (intercellular adhesion molecule-1 (ICAM-1), vascular endothelial growth factor (VEGF), von Willebrand Factor (vWF), angiopoietin (Ang)-1/2) and alveolar epithelium injury (receptor for advanced glycation end products (RAGE), surfactant protein (SP)-D, amphiregulin) biomarkers, were quantified in BAL fluid supernatant and serum and expressed in fluorescence intensities and concentrations (pg/mL).

### Data presentation and statistical analysis

Continuous variables are reported as median [1st–3rd quartiles] or mean ± standard deviation (SD), and compared using the unpaired Student t test or the Mann–Whitney test, as appropriate. Comparison of paired quantitative variables was performed using the Wilcoxon matched-pairs signed-rank test or two-way ANOVA with repeated measures when more than two groups were compared. Correlations between continuous variables were assessed using the Spearman method. Qualitative variables are expressed as numbers and percentages and compared with the Chi^2^ or Fischer tests, as recommended. Uni- and multivariable logistic regression models were used to assess the relationship between BAL fluid-to-serum concentration ratios of biomarkers, BAL fluid-to-blood ratio of monocytic HLA-DR or T CD8^+^ lymphocyte PD-1 expression, as continuous variables, and hospital mortality (dependent variable). Adjusted analyses were performed including major prognostic variables defined a priori (i.e., SOFA score [38] and driving pressure [39]). No imputation of missing variable was performed. A *p* value < 0.05 was considered significant. Statistical analyses were performed using GraphPad Prism (version 8.0, GraphPad Software, Inc., San Diego, CA, USA) and R 3.1.2 (The R Foundation for Statistical Computing, Vienna, Austria).

## Results

### Initial presentation and outcomes of patients with pneumonia-related ARDS

One hundred and eighty-eight patients with moderate-to-severe pneumonia-related ARDS were admitted to the ICU during the four-year study period, of whom 118 had non-inclusion criteria and 70 were included in the study (Additional file 1: Figure S2). A microbiological documentation was obtained in 87% (*n* = 61/70) of included patients, 67% (*n* = 47/70) of whom had bacterial infections, and 26% (*n* = 18/70) had viral infections (four had bacterial and viral coinfections) (Additional file 1: Table S2). The comorbidities, clinical and biological characteristics of patients at ICU admission and at the time the first BAL was sampled (i.e., after a median delay of one day following intubation) are presented in Additional file 1: Table S1.

In-hospital mortality was 21.4% (*n* = 15/70). Patients who were dead at hospital discharge did not exhibit more frequent ventilator-associated pneumonia episodes or septic shock during hospital stay but required more frequent renal replacement therapy than those who survived (Table 1).

**Table 1:** Characteristics and outcomes of patients with pneumonia-associated acute respiratory distress syndrome (*n *= 70) who survived (*n* = 55) to hospital discharge or not (*n* = 15)

Variables	Available data	Survived *N* = 55	Died *N* = 15	*p* value
*Demographics and comorbidities*				
Age	70	59 [51–69]	70 [57–79]	0.197
Gender, male	70	38 (69)	12 (80)	0.407
BMI, kg/cm^2^	70	27.3 [23.7–31.8]	26.4 [23.7–28.0]	0.440
Diabetes mellitus	70	16 (29)	3 (20)	0.483
COPD	70	10 (18)	3 (20)	0.872
Chronic heart failure	70	10 (18)	3 (20)	0.872
Liver cirrhosis	70	3 (5)	0 (0)	0.355
Smoker	70	24 (44)	8 (53)	0.504
*Patients characteristics upon ICU admission*				
SOFA	70	9 [7–10]	12 [5–14]	0.142
SAPS II	70	43 [31–55]	53 [41–62]	0.165
Admission-intubation^a^, days	70	0.0 [0.0–1.0]	0.0 [0.0–1.0]	0.961
Number of ARDS risk factors	70	1 [1–2]	1 [1–1]	0.239
Temperature	70	38.5 [38.0–39.1]	38.2 [37.5–39.5]	0.492
ARDS severity (Berlin)	70			0.719
*Mild*		2 (4)	0 (0)	
*Moderate*		20 (36)	5 (33)	
*Severe*		33 (60)	10 (67)	
Driving pressure, cmH_2_O	70	14 12–17]	16 [13–20]	0.240
Crs, mL/cmH_2_O	70	26 [23–34]	28 [21–33]	0.901
Lung injury score	70	2.5 [2.2–3.0]	2.2 [2.0–2.7]	0.418
PEEP, cmH_2_O	70	10 [6–12]	8 [5–10]	0.162
Tidal volume, mL/kg of PBW	70	6.1 [5.8–6.5]	6.3 [6.0–6.9]	0.642
PaO_2_/FiO_2_ ratio, mmHg	70	92 [73–139]	91 [73–188]	0.858
PaCO_2_, mmHg	70	46 [40–51]	50 [33–59]	0.858
pH	70	7.34 [7.27–7.42]	7.30 [7.25–7.39]	0.261
Arterial blood lactates, mM	70	1.5 [1.0–2.5]	1.9 [1.0–3.7]	0.410
Creatinine, µmol/L	70	85 [68–149]	130 [81–234]	0.119
Bilirubin, µmol/L	70	11 [6–18]	19 [12–58]	**0.008**
Prothrombin time, %	70	76 [64–84]	60 [42–74]	**0.018**
WBC counts, 10^3^/mm^3^	70	11.7 [8.0–19.5]	13.8 [8.3–18.6]	0.949
Neuromuscular blockers	70	40 (73)	12 (80)	0.568
Prone position	70	20 (36)	3 (20)	0.232
ECMO	70	2 (4)	1 (7)	0.607
Shock^b^	70	14 (25)	7 (47)	0.112
*Patients characteristics upon BAL 1 sampling*				
SOFA	70	8 [6–10]	11 [7–13]	0.083
Intubation-BAL 1 ^c^, days	70	1.0 [1.0–1.0]	1.0 [1.0–1.0]	0.868
BAL cells, 10^3^/mL	70	415 [224–962]	565 [250–1700]	0.657
BAL macrophages, %	70	25 [12–49]	26 [8–51]	0.775
BAL lymphocytes, %	70	3 [1–6]	5 [1–9]	0.530
BAL neutrophils, %	70	69 [38–83]	72 [38–90]	0.616
Driving pressure, cmH_2_O	70	12 [10–14]	15 [12–17]	**0.030**
Crs, mL/cmH_2_O	70	33 [26–42]	32 [22–40]	0.371
Lung injury score	70	2.5 [2.2–3.0]	2.2 [2.0–2.7]	0.418
PEEP, cmH_2_O	70	10 [8–14]	8 [5–10]	**0.018**
Tidal volume, mL/kg of PBW	70	6.1 [5.8–6.5]	6.1 [5.8–6.3]	0.647
PaO_2_/FiO_2_ ratio, mmHg	70	168 [116–241]	138 [117–238]	0.457
PaCO_2_, mmHg	70	44 [38–47]	42 [38–57]	0.931
pH	70	7.39 [7.32–7.44]	7.35 [7.26–7.40]	0.127
Arterial blood lactates, mM	70	1.3 [0.8–2.0]	1.5 [1.2–2.7]	0.175
Creatinine, µmol/L	70	91 [65–166]	179 [91–255]	**0.009**
Bilirubin, µmol/L	70	11 [7–19]	21 [11–48]	**0.029**
Prothrombin time, %	70	73 [61–80]	51 [40–66]	**0.007**
Platelets, 10^3^/mm^3^	70	198 [149–268]	189 [130–238]	0.229
WBC counts, 10^3^/mm^3^	70	10.7 [8.6–19.7]	15.5 [10.3–20.6]	0.383
Lymphocytes, 10^3^/mm^3^	62	0.8 [0.6–1.2]	1.0 [0.6–2.1]	0.366
Monocytes, 10^3^/mm^3^	62	0.5 [0.3–1.0]	0.5 [0.2–1.4]	0.908
Neutrophils, 10^3^/mm^3^	62	9.1 [7.0–17.1]	13.2 [9.8–15.3]	0.539
Neuromuscular blockers	70	36 (65)	11 (73)	0.565
Prone position	70	22 (40)	3 (20)	0.152
ECMO	70	3 (5)	3 (20)	0.074
Shock^c^	70	12 (22)	7 (47)	0.055
*Outcomes*				
VAP	70	17 (31)	5 (33)	> 0.99
Viral reactivation	70	9 (16)	5 (33)	0.161
*CMV*		2 (4)	1 (7)	0.521
*HSV*		7 (13)	4 (29)	0.215
Shock dose steroids	70	17 (31)	7 (47)	0.254
Shock^d^	70	39 (71)	9 (60)	0.420
Renal replacement therapy	70	14 (25)	9 (60)	**0.012**
ECMO	70	4 (7)	4 (27)	0.058
MV duration, days	70	8 [5–20]	9 [4–30]	0.662
MV free days at day 28, days	70	20 [11–23]	0 [0–0]	< 0.0001
ICU length of stay, day*s*	70	16 [9–25]	10 [5–33]	0.226
Hospital length of stay, days	70	24 [13–51]	10 [5–50]	0.075
ICU mortality	70	0 (0)	13 (87)	** < 0.0001**

*ARDS* acute respiratory distress syndrome, *BAL* bronchoalveolar lavage, *BMI* body mass index, *CMV* cytomegalovirus; *COPD* chronic obstructive pulmonary disease, *Crs* compliance of the respiratory system, *VAP* ventilator-acquired pneumonia, *ECMO* extracorporeal membrane oxygenation, *HSV* Herpes simplex virus, *ICU* intensive care unit, *MV* mechanical ventilation, *PEEP* positive end-expiratory pressure, *PBW* predicted body weight, *SOFA* sequential organ failure assessment, *SAPS II* simplified acute physiology score II, *WBC* white blood cell

Bolded *p* values are statistically significant (*p* < 0.05)

*p* values come from the Mann–Whitney test; categorical variables are shown as n (%); *p* values come from the Chi^2^ or the Fisher exact test, as appropriate

^a^Time elapsed between ICU admission and tracheal intubation

^b^As defined by the Sepsis 3 definition

^c^Time elapsed between tracheal intubation and sampling of the first bronchoalveolar lavage (BAL 1)

^d^As defined by the Sepsis 3 definition; continuous variables are presented as median [1^st^-3^rd^ quartiles]

### Biomarkers in the bronchoalveolar lavage fluid and serum of patients with pneumonia-related ARDS

**Fig. 1:**
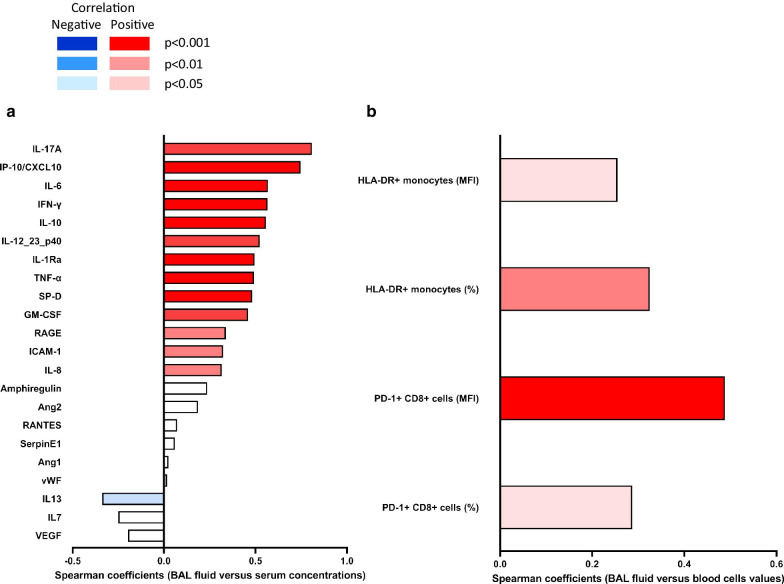
Spearman correlation coefficients of inflammatory cytokines, epithelial/endothelial injury biomarkers (**a**) and cell surface biomarkers (**b**) measured in the alveolar (bronchoalveolar lavage (BAL) fluid) and blood compartment. Positive correlations are indicated in red, negative ones in blue

**Fig. 2:**
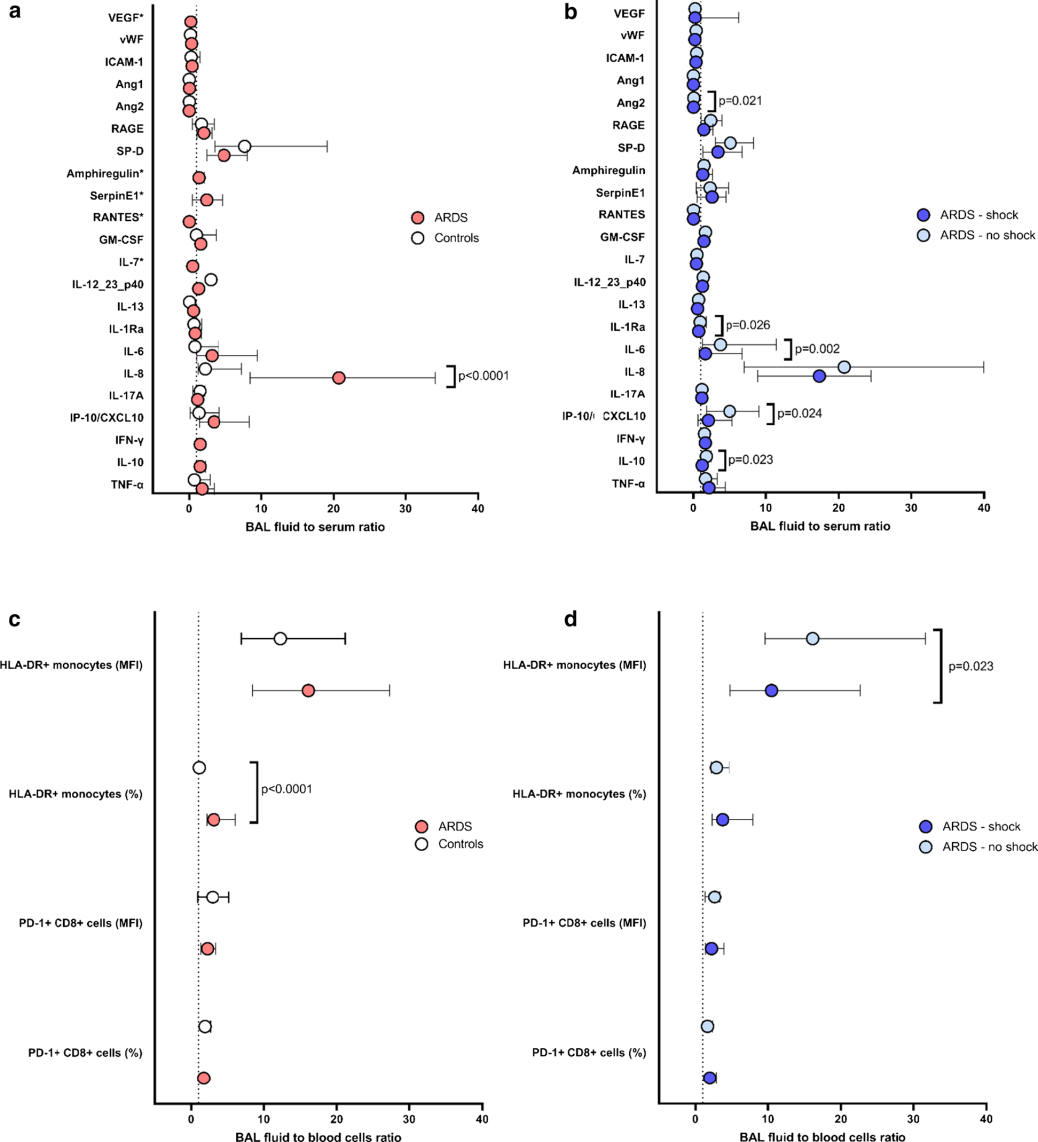
Bronchoalveolar lavage (BAL) fluid-to-serum concentration ratios (**a**, **b**) and BAL fluid-to-blood cells ratio (**c**, **d**). ARDS patients (light red) are compared with controls (opened circles) (**a**, **c**); ARDS patients with shock (dark blue) are compared with ARDS patients without shock (light blue) (**b**, **d**). Symbols indicate median and bars show the 1st and 3rd tertiles. *p* values come from the Mann–Whitney test; *Concentrations of Serpin, RANTES, IL-7, VEGF and amphiregulin could not be measured in controls; BAL fluid-to-serum concentration ratios of IFN-γ and IL-10 could not be computed because serum concentrations equaled to zero in controls

Biomarkers previously shown to be associated with key pathways involved in the pathophysiology of ARDS [8, 10] were quantified in BAL fluid and serum samples obtained in average one day after intubation and compared with those of controls. As expected, dramatically higher concentrations of these biomarkers were observed in ARDS patients (Additional file 1: Figures S3a and S3b). Significant positive correlations were observed between BAL fluid and serum concentrations for most of the studied biomarkers (Fig. 1a). In an attempt to assess the alveolar concentrations of these biomarkers relative to those of serum, we computed BAL fluid-to-serum concentration ratios (Figs. 2a, b). Strikingly, BAL fluid-to-serum ratios of most of the measured biomarkers yielded values close to 1 in ARDS and controls, indicating no concentration gradient between the alveolar and blood compartments, while values greater than one were observed for SP-D, IL-6, IL-8 and IP-10/CXCL10. Of note, the only cytokine that showed a significantly higher ratio in ARDS patients than in controls was IL-8 (*p* < 0.0001, Fig. 2a), with measured concentrations which were 20 times as high in BAL fluid than in serum. We further investigated whether patients who had septic shock at the time the biomarkers were drawn exhibited different BAL fluid-to-serum ratios than others. Interestingly, most of the cytokines involved in innate immunity (i.e., IL-1Ra, IL-6, IP-10/CXCL10 and IL-10), together with Ang2, a biomarker of endothelial injury, showed significantly higher ratios in non-shocked versus shocked patients, indicating less alveolar compartmentalization of these biomarkers in shocked patients (Fig. 2b).

An exploratory analysis assessing the prognostic value of the BAL fluid-to-serum ratio of these biomarkers indicated that IL-10, IL1-Ra, amphiregulin and RAGE were significantly associated with hospital mortality (Table 2). Yet, the only biomarker whose BAL fluid-to-serum ratio remained significantly associated with mortality after adjusting for admission SOFA and driving pressure was IL-1Ra (Table 2). A comparison of the area under the curves of receiver operating characteristic curves for serum versus BAL fluid versus BAL fluid-to-serum ratio of IL-10, IL1-Ra, amphiregulin and RAGE and hospital mortality consistently showed that BAL fluid-to-serum ratios had the strongest association with hospital mortality, except for serum RAGE levels that showed the same prediction performances than did BAL fluid-to-serum ratios (Additional file 1: Figure S4). Raw BAL fluid and serum biomarkers concentrations in survivors and decedents are shown in Additional file 1: Table S3.

**Table 2:** BAL fluid-to-serum concentration ratios of cytokines and epithelial/endothelial injury biomarkers in pneumonia-associated ARDS patients who survived (*n* = 55) to hospital discharge or not (n = 15)

	Survived (n = 55)	Died (*n* = 15)	Univariable analysis		Multivariable analysis	
			OR 95% CI	*p* value^a^	OR 95% CI	Adjusted *p* value^b^
TNF-α	1.78 [1.34–3.48]	2.17 [1.01–4.41]	0.96 [0.82–1.12]	0.612		–
IL-10	1.59 [1.13–2.39]	1.08 [0.75–0.55]	0.31 [0.11–0.87]	**0.014**	0.39 [0.14–1.09]	0.060
IFN-γ	1.53 [1.33–1.93]	1.39 [1.16–1.81]	0.78 [0.37–1.64]	0.310		–
IP-10 CXCL10	4.52 [1.73–9.52]	2.16 [0.76–4.97]	0.92 [0.81–1.04]	0.073		–
IL-17a	1.16 [1.05–1.29]	1.14 [1.07–1.21]	0.81 [0.40–1.65]	0.843		–
IL-8 CXCL8	20.96 [9.07–43.21]	11.57 [7.70–24.03]	0.97 [0.93–1.01]	0.163		–
IL-6	3.48 [1.10–10.16]	1.36 [0.61–4.72]	0.95 [0.86–1.04]	0.099		–
Il1-Ra	1.03 [0.55–1.82]	0.50 [0.29–0.72]	0.15 [0.03–0.72]	**0.006**	0.18 [0.04–0.88]	**0.036**
IL-13	0.61 [0.38–0.82]	0.57 [0.13–1.27]	0.83 [0.28–2.47]	0.805		–
SP-D	4.83 [2.57–8.55]	4.87 [1.42–6.50]	0.94 [0.82–1.07]	0.303		–
GMCSF	1.60 [1.17–2.06]	1.50 [1.14–2.45]	1.33 [0.74–2.39]	0.899		–
Amphiregulin	1.48 [1.18–2.14]	1.13 [0.92–1.45]	0.31 [0.07–1.37]	**0.037**	0.44 [0.13–1.49]	0.129
IL12-23p40	1.34 [1.16–1.59]	1.15 [1.02–1.42]	0.64 [0.18–2.25]	0.130		–
Ang2	0.04 [0.02–0.12]	0.01 [0.00–0.01]	0.01 [0.00–31.68]	0.274		–
Ang1	0.01 [0.00–0.01]	0.01 [0.00–0.08]	387 [0–575 769]	0.763		–
ICAM1	0.44 [0.10–1.03]	0.23 [0.12–0.54]	0.22 [0.04–1.19]	0.079		–
vWF	0.38 [0.17–0.44]	0.19 [0.14–0.31]	0.04 [0.00–4.43]	0.100		–
RAGE	2.36 [1.25–3.85]	1.30 [0.64–2.00]	0.58 [0.34–0.98]	**0.021**	0.59 [0.34–1.04]	0.052
VEGF	0.17 [0.07–0.74]	0.33 [0.19–1.72]	0.99 [0.91–1.07]	0.223		–
IL-7	0.48 [0.35–0.62]	0.41 [0.30–0.72]	0.76 [0.03–18.06]	0.645		–
RANTES	0.02 [0.01–0.05]	0.06 [0.01–0.24]	235 [1–66, 569]	0.178		–
SerpinE1	2.42 [0.51–4.56]	1.60 [0.29–5.66]	1.03 [0.85–1.25]	0.564		–

Variables are expressed as median [1st–3rd quartiles] of fluorescence intensity

*OR 95% CI* odds ratio and their 95% confidence interval

^a^Unadjusted *p* values come from the Mann–Whitney test

^b^Adjusted *p* values yielding statistical significance at the *p* < 0.05 level come from multivariable logistic regression analyses and were adjusted for SOFA and driving pressure by multivariable logistic regression analysis; bolded results are significant at the *p* < 0.05 level

### Cell surface biomarkers on bronchoalveolar and blood leukocytes of patients with pneumonia-related ARDS

As expected in pneumonia-related ARDS, BAL fluid cellularity was elevated (median: 470 × 10^3^ cells/mL [227–975] and differential cell counts showed a majority of neutrophils (69% [38–84], Table 1), consistent with alveolar inflammation.

**Fig. 3:**
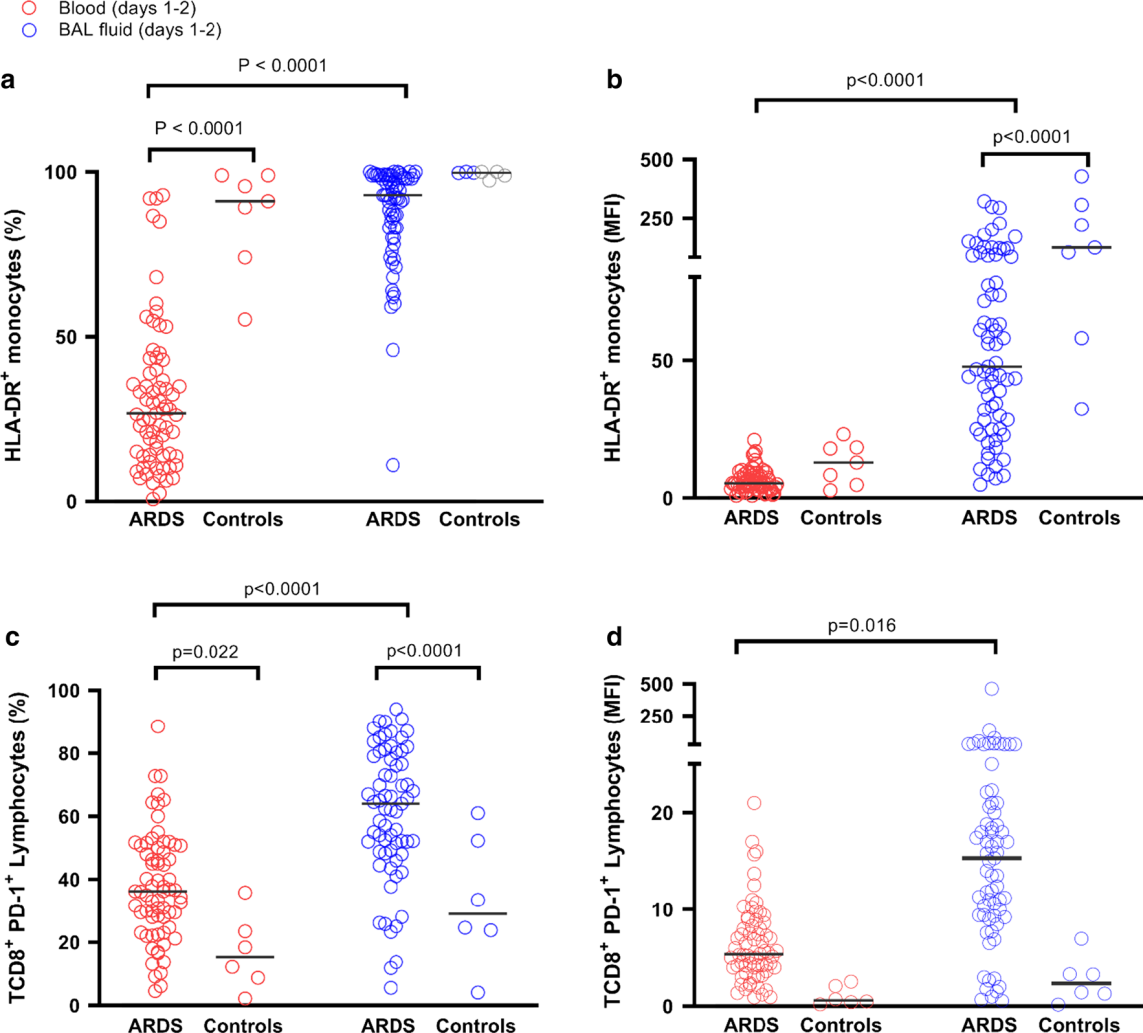
HLA-DR^+^ monocytes and T CD8 + PD-1^+^ lymphocytes in bronchoalveolar lavage fluid and blood of patients with pneumonia-related acute respiratory distress syndrome (ARDS) (*n* = 70) and controls (*n* = 7). Expression of monocytic HLA-DR was quantified in percentage of positive cells (**a**) or in mean fluorescence intensity (MFI, **b**); by two-way ANOVA with repeated measures, there was a significant effect of group (ARDS vs controls, *p* < 0.0001) and of sample compartment (BAL fluid vs blood, *p* < 0.0001), with significant interaction (group x compartment, *p* < 0.0001 in percentage and *p* = 0.0011 in MFI), on the expression of monocyte HLA-DR. Expression of PD-1 on CD8^+^ lymphocytes was quantified in percentage of positive cells (**c**) or in mean fluorescence intensity (MFI, **d**). By two-way ANOVA with repeated measures, there was a significant effect of group (ARDS vs controls, *p* < 0.001) and of sample compartment (BAL fluid vs blood, *p* < 0.001), without significant interaction (group × compartment, *p* = 0.549), when expressed in percentage of positive cells (**c**). When results were expressed in MFI (**d**), there was no significant effect of group (ARDS vs control patients, *p* = 0.252), or of sample compartment (BAL fluid vs blood, *p* = 0.404), without significant interaction (group × compartment, p = 0.488). Displayed *p* values come from post hoc comparisons performed using the Sidak’s test. Horizontal bars represent median values

We quantified the monocytic expression of HLA-DR, a prognostic cell surface biomarker in septic shock patients [15], on bronchoalveolar and circulating monocytes, within 48 h of tracheal intubation. As compared with control patients, those with pneumonia-related ARDS exhibited significantly lower HLA-DR expression, both on circulating (*p* < 0.0001 when expressed in percentage of positive cells; Fig. 3a) and alveolar (*p* < 0.0001 when expressed in MFI; Fig. 3b) monocytes. ARDS patients also displayed dramatically higher HLA-DR expression, expressed both in percentage of positive cells (*p* < 0.0001; Fig. 3a) and MFI (*p* < 0.0001; Fig. 3b), on their alveolar than on their blood monocytes, consistent with the recruitment of activated monocytes in the infected lungs. Of note, there was a significant positive correlation between HLA-DR expression on circulating and on alveolar monocytes (Fig. 1b). The BAL fluid-to-blood ratio of HLA-DR monocytic expression was computed so that to better assess the compartmentalization of this biomarker during pneumonia-associated ARDS: when expressed in percentage of HLA-DR positive monocytes, the ratio was higher in ARDS patients than in controls. We also compared this ratio between patients with and without septic shock, as HLA-DR monocytic expression is an outcome biomarker in this specific group of patients, and observed that it was lower in the former than in the latter (Fig. 2b).

There was no statistically significant association between HLA-DR expression on alveolar monocytes and hospital mortality, even after adjusting for potentially confounding variables (i.e., SOFA score and driving pressure, Additional file 1: Table S4). There was also no significant relationship between the BAL fluid-to-blood ratio of HLA-DR monocytic expression and hospital mortality. There was a negative correlation between HLA-DR on alveolar monocytes and the SOFA score (Spearman *r* = − 0.42; *p* = 0.0003).

Patients with pneumonia-related ARDS exhibited significantly higher PD-1 expression on both alveolar (*p* = 0.001) and blood (*p* = 0.022) T CD8^+^ lymphocytes than did control patients. Among ARDS patients, a higher expression of PD-1 was also observed on alveolar than on blood T CD8^+^ lymphocytes (*p* < 0.0001; Fig. 3c and *p* = 0.016; Fig. 3d), consistent with the recruitment of activated CD8^+^ lymphocytes at the site of infection. There was no statistically significant association between PD-1 on alveolar T CD8^+^ lymphocytes or the BAL fluid-to-blood ratio of PD-1^+^ CD8^+^ cells (in percentage or MFI) and hospital mortality (Additional file 1: Table S5).

## Discussion

The current study included 70 patients with pneumonia-related ARDS and quantified the concomitant concentration/cell surface expression of biomarkers in the bronchoalveolar and blood compartments. This was a cohort of homogeneous immunocompetent patients, all diagnosed with moderate-to-severe ARDS since less than 48 h when included in the study. The main results of the current study are as follows: (1) IL-8 had the highest BAL fluid-to-serum concentration ratio and IL-1Ra, IL-6, IP-10/CXCL10 and IL-10 showed higher lung/blood concentration gradients in non-shocked than in shocked patients; ((2) in an exploratory analysis, IL-1Ra were associated with hospital mortality after adjusting for major confounding variables defined a priori (i.e., SOFA and driving pressure); and (3) HLA-DR expression measured within 48 h of intubation on monocytes and PD-1 expression on T CD8^+^ lymphocytes showed a lung compartmentalization, but were not associated with hospital mortality.

The identification of reliable biomarkers constitutes a major area of research in ARDS to help predict its development, stratify disease severity into more accurate phenotypes, provide new insights into its pathogenesis and monitor response to treatment [8]. Although improvements regarding patient phenotyping have been made using multiparametric approaches combining clinical and biological variables [6, 7], no single biomarker obtained from blood samples has been shown to be consistently associated with outcomes [10], possibly because of a compartmentalization of biomarkers during pneumonia-related ARDS. In the current study, we explored the interrelation between alveolar and blood concentrations of biomarkers previously associated with ARDS and observed significant correlations between both compartments for most of the cytokines measured. Yet, alveolar concentrations of pro-inflammatory cytokines, including IL-6, IL-8 and IP-10/CXCL10, and of SP-D, were significantly higher than their serum concentrations, consistent with a lung borne production of these biomarkers, the most compartmentalized of which was IL-8, a potent neutrophil chemoattractant, confirming its pivotal role in ARDS pathophysiology [5, 9]. Moreover, the fact that patients with shock had lower BAL fluid-to-serum concentrations ratios of the main pro/anti-inflammatory cytokines (i.e., IL1-Ra, IL-6, IP-10/CXCL10 and IL-10) suggests that less lung-compartmentalization of these mediators might be a mechanism leading to extra-pulmonary organ failures complicating the course of ARDS, as previously hypothesized [40, 41]. The fact that lower values of the BAL fluid-to-serum ratio of IL-1Ra was associated with hospital mortality, even after adjusting for SOFA and driving pressure, reinforces this hypothesis.

HLA-DR expression on alveolar monocytes of ARDS patients was lower than that of control patients, suggesting a down-regulation of HLA-DR expression in the infected lungs. Such finding mirrors the previously reported down-regulation of HLA-DR expression on circulating monocytes of patients with septic shock [13]. During septic shock, monocyte deactivation, defined as diminished antigen-presenting capacity reflected by the down-expression of HLA-DR, has been repeatedly associated with morbidity and mortality [14, 15]. The decrease in HLA-DR expression on circulating monocytes is thus a robust predictor of outcome in septic shock patients, which can be restored by immunostimulation with GM-CSF [24]. However, we did not observe a significant association between early HLA-DR expression on alveolar monocytes and hospital mortality. Few studies focused on the outcome impact of a decreased alveolar monocytic HLA-DR expression. Making the hypothesis that reversing HLA-DR down-regulation on alveolar monocytes would improve outcomes, Herold et al. administrated inhaled GM-CSF in six patients with pneumonia-related ARDS with documented decreased HLA-DR expression on alveolar monocytes, as a compassionate intervention [42]. In this pilot study, inhaled GM-CSF administration was associated with improved oxygenation and restored HLA-DR expression on alveolar monocytes, but the lack of control arm and the low number of patients treated precluded any firm conclusion to be drawn. Our data show that monitoring HLA-DR expression on alveolar monocytes during the first 48 h of pneumonia-related ARDS did not allow for identifying a subset of patients at higher risk of poor outcomes, thus suggesting this biomarker should not be used—at least during the early phase of ARDS—to monitor regional immune status or guide therapeutic interventions.

Interestingly, HLA-DR expression was higher on alveolar than on circulating monocytes in pneumonia-related ARDS patients. Such compartmentalization of HLA-DR expression has already been observed in septic shock patients [43]. The fact that alveolar monocytic HLA-DR expression was also lower in ARDS than in control patients is consistent with the recruitment of circulating monocytes into the alveolar space [44]. As expected, the SOFA score was negatively correlated with HLA-DR expression on alveolar monocytes, suggesting that the number of organ failures was associated with monocyte deactivation in the lungs, as previously shown in circulating monocytes of septic shock patients [14].

We also quantified PD-1 expression on alveolar and blood T CD8^+^ lymphocytes. Patients with pneumonia-related ARDS exhibited significantly higher PD-1 expression on both alveolar and peripheral circulating T CD8^+^ lymphocytes than control patients. This is consistent with the work of Zhang et al. [45] reporting higher PD-1 expression on peripheral T cells of septic shock patients than on those of controls. Several studies reported that patients with septic shock and high levels of PD-1 expression on peripheral T lymphocytes were more likely to have an increased mortality and more occurrence of nosocomial infections [20] and Morrell et al. reported that PD-L1/PD-1 pathway-associated genes were significantly decreased in alveolar macrophages from ARDS patients who died or had prolonged mechanical ventilation [46]. However, we observed no significant association between PD-1 expression level on alveolar T CD8^+^ lymphocytes and outcomes. Additionally, patients with pneumonia-related ARDS had significantly higher PD-1 expression on alveolar than on blood T CD8^+^ lymphocytes. Such compartmentalization of PD-1 expression was already observed in preclinical experimental as well as in autopsy studies and may chiefly reflect the recruitment of activated lymphocytes at the site of infection [22, 47].

Our study certainly has a number of limitations. This is a monocentric study including a homogeneous population of patients with pneumonia-related ARDS, thus limiting its external validity and the generalizability of the findings. The relatively small number of patients included precluded validating our results in an independent validation cohort, and the results of the conducted analyses, some of which would loss statistical significance after accounting for multiple testing, should be considered exploratory and interpreted with caution. Regarding the analysis of the relationship between biomarkers and hospital mortality, we have chosen not to control all statistical tests performed for multiple testing but instead preferred to adjust for prognostic variables defined a priori (i.e., SOFA score and driving pressure). Our control patients’ population only included spontaneously breathing patients, not receiving antibiotics at the time of BAL fluid sampling, which might have contributed to between group differences. Other limitations of our study are the constraints associated with measuring BAL fluid-to-serum ratios of biomarkers, and limiting their analysis in “real life” conditions. We thus acknowledge the current study is more likely to have an impact on our understanding of the pathophysiology of the compartmentalization of biomarkers during ARDS than on clinical management. The flow cytometry gating strategy used for distinguishing alveolar monocytes from macrophages did not use antibodies for CD206 and CD169 [48] but identified side scatter intermediate (SSC), CD45^+^ and CD14^+^ cells. Although such methods were previously reported [49], we cannot exclude that our alveolar monocytes population was contaminated by macrophages. Last, we chose not to normalize BAL fluid concentrations of the studied biomarkers using BAL fluid-to-serum urea or albumin concentration ratios, as none of these methods has been shown to improve the accuracy of the measurements performed [50, 51]. Our study also has some strengths, including a prospective design allowing for uniform timing of measurements at a clinically relevant time-point and the combination of clinical, flow cytometry and cytokines data.

## Conclusion

In conclusion, this study showed that, in patients with pneumonia-associated ARDS, IL-8 was the most compartmentalized cytokine and that lower BAL fluid-to-serum concentration ratios of IL-1Ra were associated with hospital mortality, even after adjusting for SOFA and driving pressure. In contrast, neither alveolar monocytic HLA-DR expression nor T CD8^+^ lymphocyte PD-1 expression were prognostic biomarkers.

## Supplementary information

**Additional file 1.** Tables S1 to S5, Figures S1 to S4.

## Data Availability

All data generated and analyzed during this study are available on demand.

## Abbreviations

ARDS: Acute respiratory distress syndrome
BAL: Bronchoalveolar lavage
HLA-DR: Human leukocyte antigen-DR
PD-1: Programmed death receptor-1
ICU: Intensive care unit

## References

[1] Bellani G, Laffey JG, Pham T, Fan E, Brochard L, Esteban A (2016). Epidemiology, patterns of care, and mortality for patients with acute respiratory distress syndrome in intensive care units in 50 countries. JAMA.

[2] National Heart, Lung, and Blood Institute Acute Respiratory Distress Syndrome (ARDS) Clinical Trials Network (2006). Efficacy and safety of corticosteroids for persistent acute respiratory distress syndrome. N Engl J Med.

[3] Perkins GD, McAuley DF, Thickett DR, Gao F (2006). The β-agonist lung injury trial (BALTI): a randomized placebo-controlled clinical trial. Am J Respir Crit Care Med.

[4] McAuley DF, Laffey JG, O’Kane CM, Perkins GD, Mullan B, Trinder TJ (2014). Simvastatin in the acute respiratory distress syndrome. N Engl J Med.

[5] Ware LB, Koyama T, Billheimer DD, Wu W, Bernard GR, Thompson BT (2010). Prognostic and pathogenetic value of combining clinical and biochemical indices in patients with acute lung injury. Chest.

[6] Calfee CS, Delucchi K, Parsons PE, Thompson BT, Ware LB, Matthay MA (2014). Subphenotypes in acute respiratory distress syndrome: latent class analysis of data from two randomised controlled trials. Lancet Respir Med.

[7] Famous KR, Delucchi K, Ware LB, Kangelaris KN, Liu KD, Thompson BT (2017). Acute respiratory distress syndrome subphenotypes respond differently to randomized fluid management strategy. Am J Respir Crit Care Med.

[8] García-Laorden MI, Lorente JA, Flores C, Slutsky AS, Villar J (2017). Biomarkers for the acute respiratory distress syndrome: how to make the diagnosis more precise. Ann Transl Med.

[9] Donnelly SC, Strieter RM, Kunkel SL, Walz A, Robertson CR, Carter DC (1993). Interleukin-8 and development of adult respiratory distress syndrome in at-risk patient groups. Lancet Lond Engl.

[10] van der Zee P, Rietdijk W, Somhorst P, Endeman H, Gommers D (2020). A systematic review of biomarkers multivariately associated with acute respiratory distress syndrome development and mortality. Crit Care Lond Engl.

[11] ARDS Definition Task Force (2012). Acute respiratory distress syndrome: the Berlin definition. JAMA.

[12] Monneret G, Lepape A, Venet F (2011). A dynamic view of mHLA-DR expression in management of severe septic patients. Crit Care.

[13] Hotchkiss RS, Monneret G, Payen D (2013). Immunosuppression in sepsis: a novel understanding of the disorder and a new therapeutic approach. Lancet Infect Dis.

[14] Landelle C, Lepape A, Voirin N, Tognet E, Venet F, Bohé J (2010). Low monocyte human leukocyte antigen-DR is independently associated with nosocomial infections after septic shock. Intensive Care Med.

[15] Monneret G, Lepape A, Voirin N, Bohé J, Venet F, Debard A-L (2006). Persisting low monocyte human leukocyte antigen-DR expression predicts mortality in septic shock. Intensive Care Med.

[16] Hotchkiss RS, Monneret G, Payen D (2013). Sepsis-induced immunosuppression: from cellular dysfunctions to immunotherapy. Nat Rev Immunol.

[17] Francisco LM, Sage PT, Sharpe AH (2010). The PD-1 pathway in tolerance and autoimmunity. Immunol Rev.

[18] Monaghan SF, Thakkar RK, Heffernan DS, Huang X, Chung C-S, Lomas-Neira J (2012). Mechanisms of indirect acute lung injury: a novel role for the co-inhibitory receptor, programmed death-1 (PD-1). Ann Surg.

[19] Brahmamdam P, Inoue S, Unsinger J, Chang KC, McDunn JE, Hotchkiss RS (2010). Delayed administration of anti-PD-1 antibody reverses immune dysfunction and improves survival during sepsis. J Leukoc Biol.

[20] Guignant C, Lepape A, Huang X, Kherouf H, Denis L, Poitevin F (2011). Programmed death-1 levels correlate with increased mortality, nosocomial infection and immune dysfunctions in septic shock patients. Crit Care.

[21] Huang X, Venet F, Wang YL, Lepape A, Yuan Z, Chen Y (2009). PD-1 expression by macrophages plays a pathologic role in altering microbial clearance and the innate inflammatory response to sepsis. Proc Natl Acad Sci.

[22] Boomer JS, To K, Chang KC, Takasu O, Osborne DF, Walton AH (2011). Immunosuppression in patients who die of sepsis and multiple organ failure. JAMA.

[23] Nierhaus A, Montag B, Timmler N, Frings DP, Gutensohn K, Jung R (2003). Reversal of immunoparalysis by recombinant human granulocyte-macrophage colony-stimulating factor in patients with severe sepsis. Intensive Care Med.

[24] Meisel C, Schefold JC, Pschowski R, Baumann T, Hetzger K, Gregor J (2009). GM-CSF to reverse sepsis-associated immunosuppression: a double-blind randomized placebo-controlled multicenter trial. Am J Respir Crit Care Med.

[25] Payen D, Faivre V, Miatello J, Leentjens J, Brumpt C, Tissières P (2019). Multicentric experience with interferon gamma therapy in sepsis induced immunosuppression. A case series. BMC Infect Dis.

[26] Pfortmueller CA, Meisel C, Fux M, Schefold JC (2017). Assessment of immune organ dysfunction in critical illness: utility of innate immune response markers. Intensive Care Med Exp.

[27] Venet F, Lukaszewicz A-C, Payen D, Hotchkiss R, Monneret G (2013). Monitoring the immune response in sepsis: a rational approach to administration of immunoadjuvant therapies. Curr Opin Immunol.

[28] Venet F, Monneret G (2018). Advances in the understanding and treatment of sepsis-induced immunosuppression. Nat Rev Nephrol.

[29] Gall J-RL, Lemeshow S, Saulnier F (1993). A New Simplified Acute Physiology Score (SAPS II) Based on a European/North American Multicenter Study. JAMA.

[30] Mercat A, Richard J-CM, Vielle B, Jaber S, Osman D, Diehl J-L (2008). Positive end-expiratory pressure setting in adults with acute lung injury and acute respiratory distress syndrome: a randomized controlled trial. JAMA.

[31] Papazian L, Forel J-M, Gacouin A, Penot-Ragon C, Perrin G, Loundou A (2010). Neuromuscular blockers in early acute respiratory distress syndrome. N Engl J Med.

[32] Griffiths MJ, Evans TW (2005). Inhaled nitric oxide therapy in adults. N Engl J Med.

[33] Guérin C, Reignier J, Richard J-C, Beuret P, Gacouin A, Boulain T (2013). Prone positioning in severe acute respiratory distress syndrome. N Engl J Med.

[34] Ferguson ND, Fan E, Camporota L, Antonelli M, Anzueto A, Beale R (2012). The Berlin definition of ARDS: an expanded rationale, justification, and supplementary material. Intensive Care Med.

[35] Bouadma L, Mourvillier B, Deiler V, Le Corre B, Lolom I, Régnier B (2010). A multifaceted program to prevent ventilator-associated pneumonia: impact on compliance with preventive measures. Crit Care Med.

[36] Mekontso Dessap A, Katsahian S, Roche-Campo F, Varet H, Kouatchet A, Tomicic V (2014). Ventilator-associated pneumonia during weaning from mechanical ventilation: role of fluid management. Chest.

[37] Meyer KC, Raghu G, Baughman RP, Brown KK, Costabel U, du Bois RM (2012). An Official American Thoracic Society clinical practice guideline: the clinical utility of bronchoalveolar lavage cellular analysis in interstitial lung disease. Am J Respir Crit Care Med.

[38] Vincent JL, Moreno R, Takala J, Willatts S, De Mendonça A, Bruining H (1996). The SOFA (Sepsis-related Organ Failure Assessment) score to describe organ dysfunction/failure. On behalf of the Working Group on Sepsis-Related Problems of the European Society of Intensive Care Medicine. Intensive Care Med.

[39] Amato MBP, Meade MO, Slutsky AS, Brochard L, Costa ELV, Schoenfeld DA (2015). Driving pressure and survival in the acute respiratory distress syndrome. N Engl J Med.

[40] Tremblay L, Valenza F, Ribeiro SP, Li J, Slutsky AS (1997). Injurious ventilatory strategies increase cytokines and c-fos m-RNA expression in an isolated rat lung model. J Clin Invest.

[41] Parsons PE, Eisner MD, Thompson BT, Matthay MA, Ancukiewicz M, Bernard GR (2005). Lower tidal volume ventilation and plasma cytokine markers of inflammation in patients with acute lung injury. Crit Care Med.

[42] Herold S, Hoegner K, Vadász I, Gessler T, Wilhelm J, Mayer K (2014). Inhaled granulocyte/macrophage colony-stimulating factor as treatment of pneumonia-associated acute respiratory distress syndrome. Am J Respir Crit Care Med.

[43] Skirecki T, Mikaszewska-Sokolewicz M, Hoser G, Zielińska-Borkowska U (2016). The early expression of HLA-DR and CD64 myeloid markers is specifically compartmentalized in the blood and lungs of patients with septic shock. Mediators Inflamm.

[44] Goto Y, Hogg JC, Whalen B, Shih C-H, Ishii H, van Eeden SF (2004). Monocyte recruitment into the lungs in pneumococcal pneumonia. Am J Respir Cell Mol Biol.

[45] Zhang Y, Li J, Lou J, Zhou Y, Bo L, Zhu J (2011). Upregulation of programmed death-1 on T cells and programmed death ligand-1 on monocytes in septic shock patients. Crit Care.

[46] Morrell ED, Wiedeman A, Long SA, Gharib SA, West TE, Skerrett SJ (2018). Cytometry TOF identifies alveolar macrophage subtypes in acute respiratory distress syndrome. JCI Insight.

[47] Erickson JJ, Gilchuk P, Hastings AK, Tollefson SJ, Johnson M, Downing MB (2012). Viral acute lower respiratory infections impair CD8+ T cells through PD-1. J Clin Invest.

[48] Yu Y-RA, Hotten DF, Malakhau Y, Volker E, Ghio AJ, Noble PW (2016). Flow cytometric analysis of myeloid cells in human blood, bronchoalveolar lavage, and lung tissues. Am J Respir Cell Mol Biol.

[49] Brittan M, Barr L, Morris AC, Duffin R, Rossi F, Johnston S (2012). A novel subpopulation of monocyte-like cells in the human lung after lipopolysaccharide inhalation. Eur Respir J.

[50] Dargaville PA, South M, Vervaart P, McDougall PN (1999). Validity of markers of dilution in small volume lung lavage. Am J Respir Crit Care Med.

[51] Marcy TW, Merrill WW, Rankin JA, Reynolds HY (1987). Limitations of using urea to quantify epithelial lining fluid recovered by bronchoalveolar lavage. Am Rev Respir Dis.

