# Supplementary material for: Alveolar compartmentalization of inflammatory and immune cell biomarkers in pneumonia-related ARDS
Source: Crit Care. 2021 Jan 9;25:23. doi: 10.1186/s13054-020-03427-y (PMC7794625; doi:10.1186/s13054-020-03427-y)

**Alveolar compartmentalization of inflammatory and immune cell biomarkers in pneumonia-related ARDS**

**Additional File 1**

Table of content

[Additional file 1: Table S1. 2](#_Toc57393554)

[Additional file 1: Table S2. 4](#_Toc57393555)

**Additional file 1: Table S3……………………………………………………………………………………………………………………….……………5**

**Additional file 1: Table S4………………………………………………………………………………………………………………………….………..6**

**Additional file 1: Table S5……………………………………………………………………………………………………………………….…………..7**

Additional file 1: Figure S1. 8

Additional file 1: Figure S2. 9

Additional file 1: Figure S3. 10

**Additional file 1: Figure S4…………………………………………………………………………………………………………………………………12**

Additional file 1: Table S1. Characteristics of control patients (n=7) included in the study

| Variables | Control patients^a^ | | | | | | | |
| --- | --- | --- | --- | --- | --- | --- | --- | --- |
|  | 1 | 2 | 3 | 4 | | 5 | 6 | 7 |
| Age | 40 | 72 | 30 | 25 | | 40 | 25 | 67 |
| Gender | Male | Male | Male | Male | | Female | Male | Male |
| Immunosuppression | No | No | No | No | | No | No | No |
| Comorbidities | None | Prostate cancer (2005, in remission) | None | None | | None | None | Ischemic heart disease, hypertension, COPD |
| Smoker | No | Yes | Yes | No | | No | No | Yes |
| Clinical indication for bronchoscopy | Micronodules in RUL | Ground glass lesions in right upper lobe + nodular lesion | Suspicion of tuberculosis | Hilar adenopathy | | Lung micronodules and positive IFN-γ release assay | Weight loss, hilar bilateral lymphadenopathy and interstitial abnormalities | Pulmonary isolated cysts in RLL in context of medical history of cerebral adenocarcinoma and smoking |
| Characteristics upon BAL sampling | | | | | | | | |
| WBC counts, 10^3^/mm^3^  Neutrophils  Lymphocytes  Monocytes | 6.4  3.5  2.4  0.4 | 17.4  14.4  2.2  0.3 | 5.0  2.4  1.5  0.6 | | 6.0  3.7  1.3  0.8 | 6.4  3.5  2.4  0.4 | 6  3.7  1.3  0.7 | 12.2  9  1.3  0.7 |
| BAL cytology  Cell counts, 10^3^/mL  Macrophages, %  Neutrophils, %  Lymphocytes, % | 77  87  1  12 | 70  84  15  1 | 197  66  1  30 | | 130  66  1  33 | 77  87  0  13 | 130  66  0.5  33 | 143.3  29  1  49 |
| Final diagnosis | Connective tissue disease | Asthma | Vasculitis | | No clear diagnosis | No clear diagnosis | Disseminated tuberculosis | Lung cancer |

^a^All control patients were free of ARDS, active pulmonary infection, infiltrative lung disease, and immunosuppression; RLL, right lower lobe; RUL, right upper lobe

Additional file 1: Table S2. Microbiological documentation of patients (n=70) with pneumonia-related ARDS

|  | N (%) |
| --- | --- |
| **Bacteria** | **47 (67 %) ^a^** |
| Enterobacteriaceae | 16 |
| Streptococcus pneumoniae | 12 |
| Staphylococcus aureus | 7 |
| Legionella pneumophila | 4 |
| Mycoplasma pneumoniae | 2 |
| Group A streptococcus | 2 |
| Haemophilus influenzae | 3 |
| **Virus** | **18 (26%) ^a^** |
| Influenza A (H1N1)pdm2009 | 11 |
| Rhinovirus | 2 |
| Respiratory syncytial virus | 2 |
| Metapneumovirus | 1 |
| Adenovirus/ Coronavirus | 3 |
| **No documentation** | **9 (13%)** |

^a^ Including four patients with bacterial-viral co-infection

Additional file 1: Table S3. Measurements of cytokines and epithelial/endothelial injury biomarkers in serum and broncho-alveolar lavage (BAL) fluid in ARDS patients who survived (n=55) or died (n=15) in the hospital.

|  | **Serum concentrations** | | | **BAL fluid concentrations** | | |
| --- | --- | --- | --- | --- | --- | --- |
| **Biomarkers^a^** | **Survived**  **(n=55)** | **Died**  **(n=15)** | **P value/adjusted p value^b^** | **Survived**  **(n=55)** | **Died**  **(n=15)** | **P value/adjusted p value^b^** |
| **TNF-α** | 27.0 [24.1-35.2] | 32.0 [24.0-45.2] | 0.390 | 48.7 [35.7-97.0] | 41.0 [27.0-130.5] | 0.396 |
| **IL-10** | 38.0 [29.0-69.0] | 55.5 [37.5-65.5] | 0.192 | 60.0 [43.0-97.6] | 62.0 [39.7-80.0] | 0.374 |
| **IFN-γ** | 29.0 [27.0-32.1] | 29.0 [18.5-33.5] | 0.867 | 42.7 [35.0-59.0] | 34.5 [24.0-46.5] | **0.042/**0.115 |
| **IP-10/CXCL10** | 387.7 [185.7-4322.9] | 1797.0 [818.5-6559.0] | 0.143 | 3255.2 [717.5-18119.2] | 3176.5 [1051.0-12385.5] | 0.937 |
| **IL-17a** | 25.0 [24.0-28.0] | 25.0 [15.0-28.0] | 0.445 | 29.2 [26.7-34.1] | 28.0 [19.2-32.7] | 0.382 |
| **IL-8** | 178.5 [107.7-436.7] | 261.0 [177.5-677.5] | 0.222 | 4520.0 [2533.7-8399.7] | 4712 [1965.5-7258.5] | 0.704 |
| **IL-6** | 400.2 [203.2-2500.9] | 1072.0 [260.0-4399.2] | 0.420 | 2596.0 [709.7-8400.1] | 2214.0 [432.5-6960.2] | 0.775 |
| **IL-1Ra** | 2995.0 [1697.7-6185.5] | 3750.0 [1501.9-7357.7] | 0.830 | 3105.5 [1671.7-6094.9] | 2017.0 [720.6-3603.0] | **0.049/**0.069 |
| **IL-13** | 33.0 [25.5-50.0] | 30.5 [16.7-66.7] | 0.470 | 19.0 [16.0-23.2] | 17.0 [14.0-20.0] | **0.042/**0.122 |
| **SP-D** | 1381.2 [621.2-2399.6] | 1338.0 [581.2-2733.5] | 0.939 | 8173.5 [4435.1-10195.0] | 4947.0 [3575.7-9236.5] | 0.260 |
| **GM-CSF** | 28.0 [24.0-31.7] | 28.0 [26.0-35.0] | 0.982 | 43.5 [31.0-62.4] | 44.0 [32.2-74.5] | 0.692 |
| **Amphiregulin** | 11.0 [10.0-12.0] | 11.0 [10.0-12.2] | 1.0 | 15.5 [12.4-24.2] | 14.0 [11.5-16.0] | 0.170 |
| **IL12-23p40** | 22.0 [20.0-23.0] | 22.0 [19.0-25.7] | 0.621 | 28.2 [24.7-35.5] | 26.0 [19.5-31.5] | 0.222 |
| **Ang2** | 2075.7 [926.5-3089.7] | 2941.0 [1705.1-5389.6] | 0.160 | 59.5 [35.7-135.8] | 67.0 [35.0-210.9] | 0.905 |
| **Ang1** | 5227.0 [3333.2-6941.1] | 3960.0 [1218.2-5513.5] | 0.091 | 27.0 [22.0-51.7] | 26.0 [20.0-39.5] | 0.396 |
| **ICAM1** | 4509.7 [3516.4-6892.6] | 4202.0 [3507.2-9696.2] | 0.490 | 1976.5 [456.7-4246.7] | 1435.0 [503.0-2460.0] | 0.401 |
| **vWF** | 84.5 [70.4-115.9] | 96.0 [83.7-124.7] | 0.254 | 29.0 [24.0 -37.2] | 24.0 [21.5-33.5] | 0.163 |
| **RAGE** | 1040.0 [685.5-1402.0] | 1421.0 [1053.5-2021.2] | **0.035/**0.207 | 2877.0 [1147.2-4176.0] | 1950.0 [632.5-3682.7] | 0.256 |
| **VEGF** | 131.7 [62.7-272.4] | 52.7 [25.4-197.7] | 0.113 | 23.5 [11.9-50.0] | 36.7 [12.1-52.1] | 0.600 |
| **IL-7** | 22.0 [17.0-30.7] | 26.0 [15.2-34.2] | 0.781 | 11.0 [11.0-12.0] | 11.0 [10.0-11.2] | 0.245 |
| **RANTES** | 2741.5 [1747.5-4311.1] | 1882.2 [334.4-3809.4] | 0.084 | 63.5 [33.6-134.2] | 42.0 [31.7-137.4] | 0.549 |
| **SerpinE1_PAI1** | 2159.0 [1707.7-3576.7] | 2858.0 [1934.1-3886.0] | 0.537 | 5690.2 [1464.7-13860.0] | 5263.0 [622.2-10730.0] | 0.480 |

Variables are expressed as median [1^st^-3^rd^ quartiles]; ^a^ All cytokine concentrations are expressed as fluorescence intensity values; ^b^ Values yielding statistical significance at the p<0.05 level were adjusted for driving pressure and SOFA; **Bolded** results are significant at the p<0.05 level.

Additional file 1: Table S4. Uni- and multiple logistic regression analyses exploring the relationship between broncho-alveolar monocytic HLA-DR expression (BAL HLA-DRm) and BAL-to-blood ratio of HLA-DRm and hospital mortality (dependent variable).

| **Independent variables** | **Univariable analysis** | | **Multivariable analysis** | |
| --- | --- | --- | --- | --- |
|  | OR (95% CI) | p | aOR (95% CI) | p |
| ***Model 1 (BAL HLA-DR_m_ expressed as a continuous variable in percentage)*** | | | | |
| BAL HLA-DR_m_ (%) | 0.99 [0.95-1.02] | 0.451 | 0.99 [0.96-1.04] | 0.955 |
| SOFA, per point | 1.15 [0.97-1.36] | 0.107 | 1.13 [0.92-1.38] | 0.237 |
| Driving pressure, per mmHg | 1.14 [0.99-1.33] | 0.074 | 1.13 [0.95-1.33] | 0.158 |
| ***Model 2 (BAL HLA-DR_m_ expressed as a continuous variable in MFI)*** | | | | |
| BAL HLA-DR_m_ (MFI) | 0.99 [0.95-1.02] | 0.451 | 1.01 [0.99-1.02] | 0.136 |
| SOFA, per point | 1.15 [0.97-1.36] | 0.107 | 1.21 [0.97-1.49] | 0.083 |
| Driving pressure, per mmHg | 1.14 [0.99-1.33] | 0.074 | 1.11 [0.95-1.31] | 0.197 |
| ***Model 3 (BAL to blood ratio of HLA-DR_m_ expressed as a continuous variable in percentage)*** | | | | |
| BAL/blood HLA-DR_m_ (%) | 0.99 [0.95-1.05] | 0.950 | 1.00 [0.95-1.06] | 0.925 |
| SOFA, per point | 1.15 [0.97-1.36] | 0.107 | 1.13 [0.94-1.37] | 0.193 |
| Driving pressure, per mmHg | 1.14 [0.99-1.33] | 0.074 | 1.13 [0.95-1.33] | 0.158 |
| ***Model 4 ( BAL to blood ratio of HLA-DR_m_ expressed as a continuous variable in MFI)*** | | | | |
| BAL/blood HLA-DR_m_ (MFI) | 1.01 [0.99-1.03] | 0.155 | 1.02 [0.99-1.04] | 0.070 |
| SOFA, per point | 1.15 [0.97-1.36] | 0.107 | 1.23 [0.99-1.28] | 0.062 |
| Driving pressure, per mmHg | 1.14 [0.99-1.33] | 0.074 | 1.08 [0.92-1.28] | 0.334 |

OR, odds ratio; CI; Confidence interval; aOR, adjusted odds ratio; MFI, mean fluorescence intensity;

Additional file 1: Table S5. Uni- and multiple logistic regression analyses exploring the relationship between broncho-alveolar CD8^+^ lymphocytes PD1 (CD8^+^ PD1^+^) expression and BAL-to-blood ratio of CD8^+^ PD1^+^ and hospital mortality (dependent variable)

| Independent variables | Univariable analysis | | Multivariable analysis | |  |
| --- | --- | --- | --- | --- | --- |
|  | **OR (95% CI)** | **p** | **aOR (95% CI)** | **p** |  |
| *Model 1 (BAL PD1 expressed as a continuous variable in percentage)* | | | | | |
| BAL PD1 (%) | 0.99 [0.96-1.02] | 0.474 | 0.99 [0.97-1.03] | >0.99 |  |
| SOFA, per point | 1.15 [0.97-1.36] | 0.107 | 1.10 [0.90-1.35] | 0.335 |  |
| Driving pressure, per mmHg | 1.14 [0.99-1.33] | 0.074 | 1.12 [0.95-1.33] | 0.171 |  |
| *Model 2 (BAL PD1 expressed as a continuous variable in MFI)* | | | | | |
| BAL PD1 (MFI) | 0.99 [0.95-1.02] | 0.451 | 0.99 [0.94-1.04] | 0.630 |  |
| SOFA, per point | 1.15 [0.97-1.36] | 0.107 | 1.10 [0.90-1.34] | 0.354 |  |
| Driving pressure, per mmHg | 1.14 [0.99-1.33] | 0.074 | 1.11 [0.94-1.30] | 0.216 |  |
| *Model 3 (BAL to blood ratio of PD1 expressed as a continuous variable in percentage)* | | | | |  |
| BAL/blood PD1^+^ CD8^+^ cells (%) | 0.99 [0.60-1.65] | 0.995 | 1.09 [0.64-1.86] | 0.740 |  |
| SOFA, per point | 1.15 [0.97-1.36] | 0.107 | 1.11 [0.91-1.35] | 0.315 |  |
| Driving pressure, per mmHg | 1.14 [0.99-1.33] | 0.074 | 1.13 [0.96-1.34] | 0.151 |  |
| *Model 4 (BAL to blood ratio of PD1 expressed as a continuous variable in MFI)* | | | | |  |
| BAL/blood PD1^+^ CD8^+^ cells (MFI) | 0.97 [0.84-1.13] | 0.738 | 0.99 [0.85-1.14] | 0.889 |  |
| SOFA, per point | 1.15 [0.97-1.36] | 0.107 | 1.13 [0.92-1.35] | 0.281 |  |
| Driving pressure, per mmHg | 1.14 [0.99-1.33] | 0.074 | 1.11 [0.94-1.30] | 0.205 |  |

OR, odds ratio; CI; Confidence interval; aOR, adjusted odds ratio; MFI, mean fluorescence intensity;

Additional file 1: Figure S1.

Gating strategy of flow cytometric analyses of monocytes and T-cell subsets in broncho-alveolar lavage fluid (A) and blood (B). The same gating strategy was used in blood and BAL fluid samples. Monocytes, and lymphocytes were first gated on a side scatter-area (SSC-A) *versus* CD45 (the leukocyte common antigen) flow cytometry dot plots. Monocytes were defined as Side Scatter (SS) intermediate, CD45^+^ and CD14^+^ cells. Expression of HLA-DR was then analyzed (upper panel in A and B). T CD8+ lymphocytes were identified as CD45^+^ CD3^+^ CD8^+^ cells within the CD45^+^ SS low lymphocyte gate. Expression of PD-1 was then analyzed on T CD8^+^ lymphocytes (lower panel in A and B). HLA-DR and PD-1 quantification was expressed in percentage or mean fluorescence of intensity (MFI).


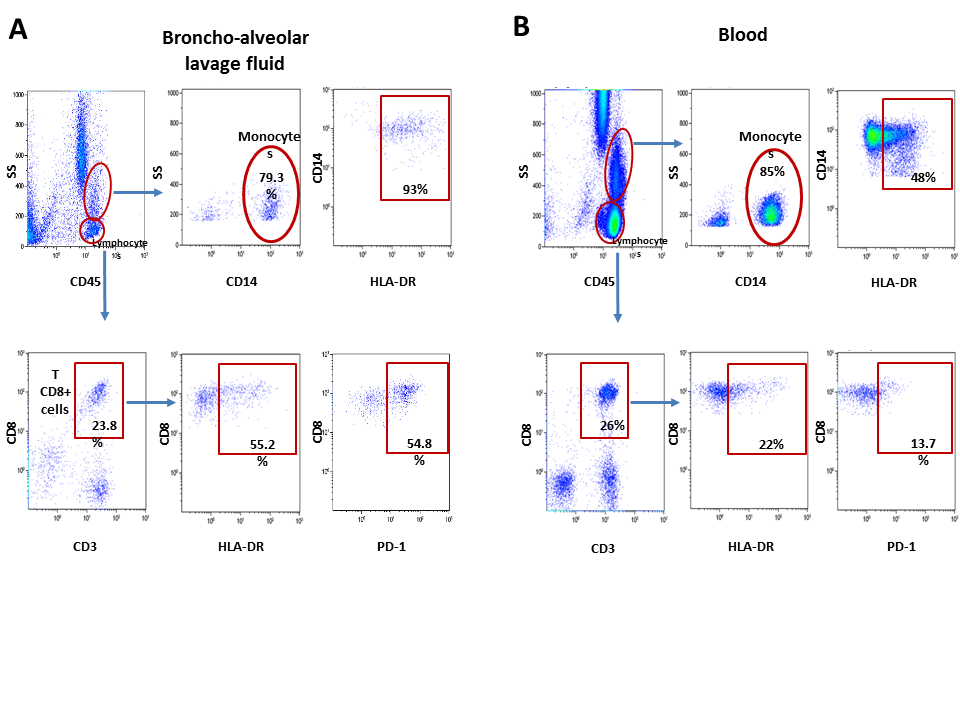


Additional file 1: Figure S2.

**Flow chart of patients with pneumonia-related moderate/severe acute respiratory distress syndrome (ARDS) included in the study.** BAL, broncho-alveolar lavage; CRD: chronic respiratory disease; LTO: long term oxygenotherapy; OTI: oro-tracheal intubation; WH/WD of LST: withholding/withdrawal of life-sustaining therapies.


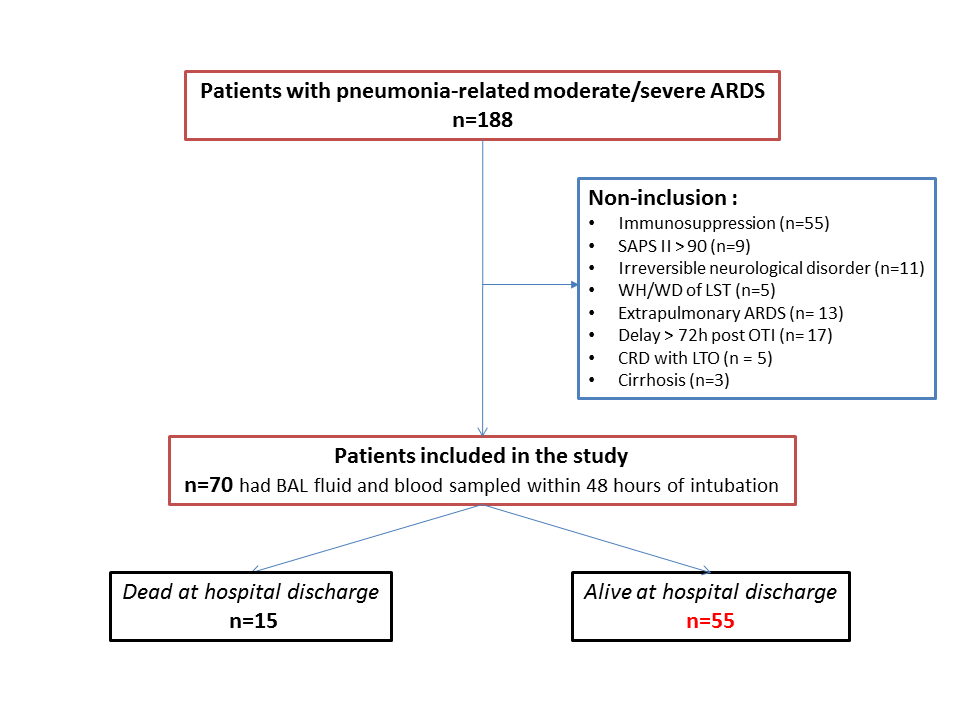


Additional file 1: Figure S3.

Serum and broncho-alveolar lavage (BAL) fluid concentrations of biomarkers associated with the acute respiratory distress syndrome (ARDS). Red circles correspond to ARDS patients (n=70) and open circles to controls (n=6). **C**oncentrations are expressed in fluorescence intensity (**A**) **or pg/mL (B; note that some of the subfigures are presented with a log scale)**. P values come from the Mann-Whitney test.

A.


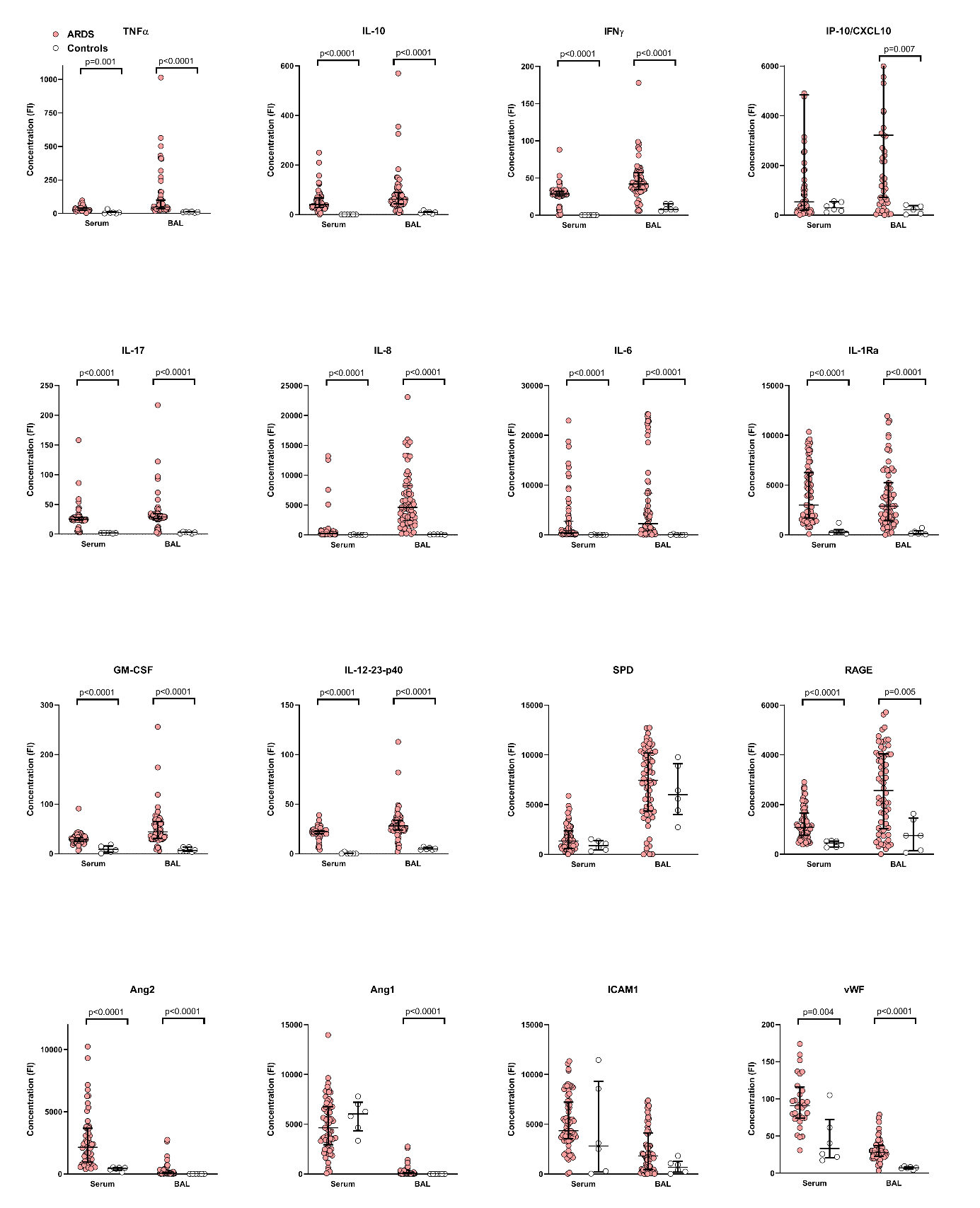


**B.**

**
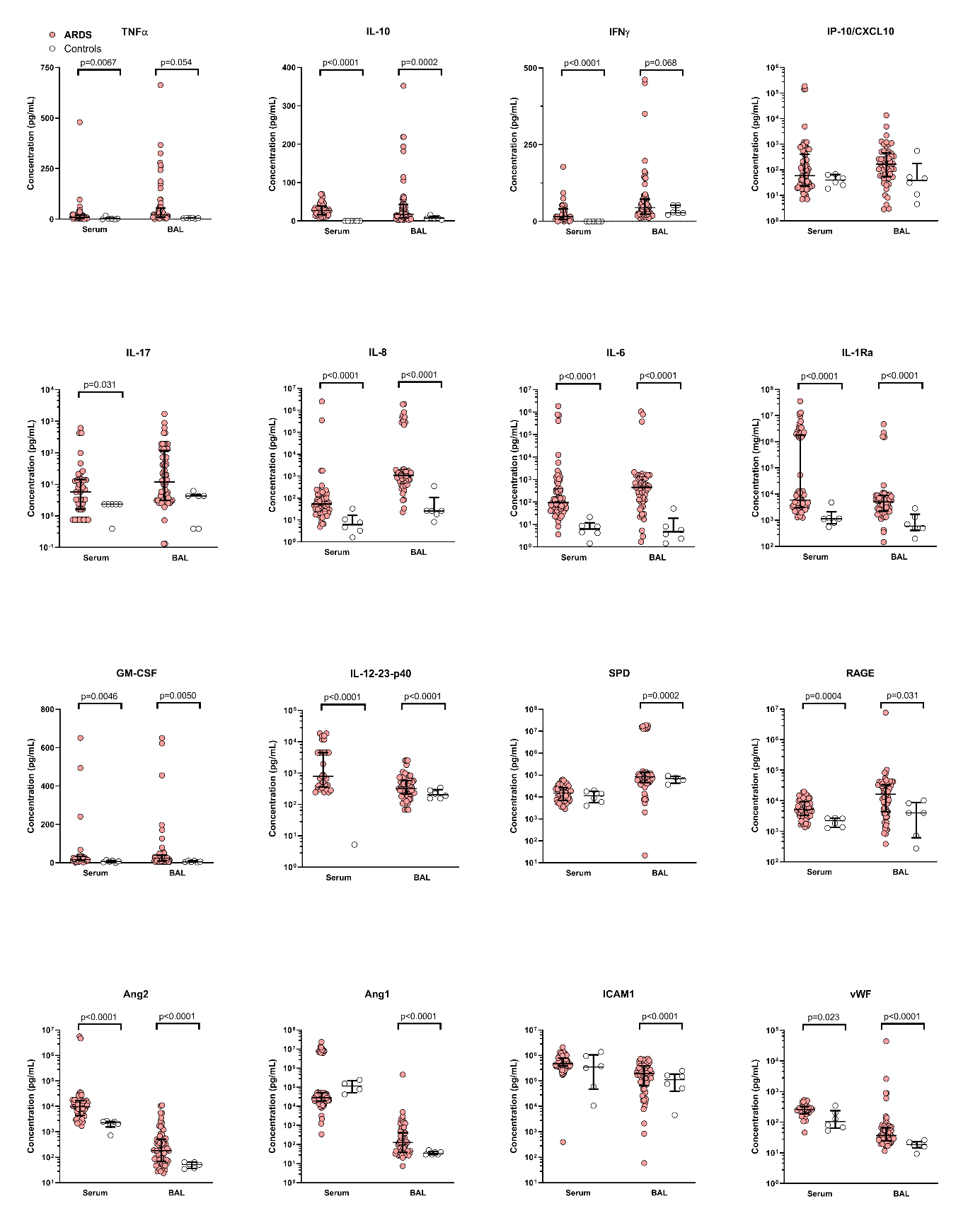
**

**Additional file 1: Figure S4.**

**Receiver Operating Characteristic (ROC) curves for biomarkers obtained in serum (left column), broncho-alveolar lavage (BAL) fluid (middle column), and BAL fluid -to-serum ratio, and hospital mortality.** A) Il-1Ra; B) IL-10; C) Amphiregulin; D) RAGE. The area under the curve (AUC) is provided and its 95% confidence interval is displayed between brackets.


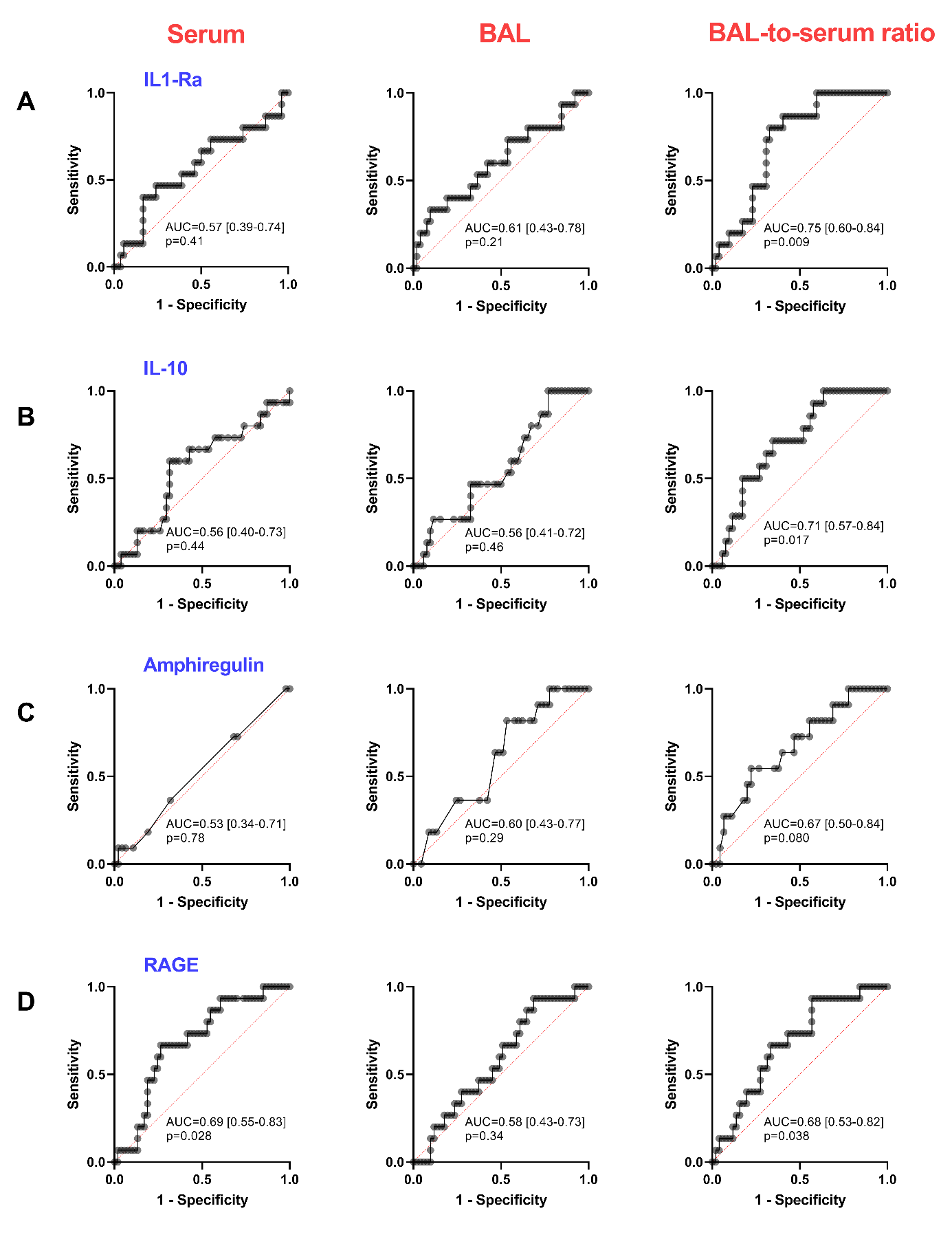

Supplement: Supplementary file 1 — Additional file 1. Tables S1 to S5, Figures S1 to S4. [file 13054_2020_3427_MOESM1_ESM.docx]
